# Identification of CDK gene family and functional analysis of *CqCDK15* under drought and salt stress in quinoa

**DOI:** 10.1186/s12864-023-09570-4

**Published:** 2023-08-17

**Authors:** Wangtian Wang, Wenyu Liu, Baoqiang Wang

**Affiliations:** 1 State Key Laboratory of Aridland Crop Science, Gansu Agricultural University, Lanzhou, 730070 China; 2 College of life science and technology, Gansu Agricultural University, Lanzhou, 730070 China; 3 Gansu Academy of Agricultural Sciences, Lanzhou, 730070 China

**Keywords:** CDK gene family, Drought and salt stress, Quinoa, Subcellular localization, Transgenic

## Abstract

as one of the oldest cultivated crops in the world, quinoa has been widely valued for its rich nutritional value and green health. In this study, 22 CDK genes (*CqCDK01-CqCDK22*) were identified from quinoa genome using bioinformatics method. The number of amino acids was 173–811, the molecular weight was 19,554.89 Da-91,375.70 Da, and the isoelectric point was 4.57–9.77. The phylogenetic tree divided 21 CqCDK genes into six subfamilies, the gene structure showed that 12 (54.5%) CqCDK genes (*CqCDK03*, *CqCDK04*, *CqCDK05*, *CqCDK06*, *CqCDK07*, *CqCDK11*, *CqCDK14*, *CqCDK16*, *CqCDK18*, *CqCDK19*, *CqCDK20* and *CqCDK21*) had UTR regions at 5’ and 3’ ends. Each CDK protein had different motifs (3–9 motifs), but the genes with the same motifs were located in the same branch. Promoter analysis revealed 41 cis-regulatory elements related to plant hormones, abiotic stresses, tissue-specific expression and photoresponse. The results of real-time fluorescence quantitative analysis showed that the expression level of some CDK genes was higher under drought and salt stress, which indicated that CDK genes could help plants to resist adverse environmental effects. Subcellular localization showed that *CqCDK15* gene was localized to the nucleus and cytoplasm, and transgenic plants overexpressing *CqCDK15* gene showed higher drought and salt tolerance compared to the controls. Therefore, CDK genes are closely related to quinoa stress resistance. In this study, the main functions of quinoa CDK gene family and its expression level in different tissues and organs were analyzed in detail, which provided some theoretical support for quinoa stress-resistant breeding. Meanwhile, this study has important implications for further understanding the function of the CDK gene family in quinoa and our understanding of the CDK family in vascular plant.

**Supplementary Information:**

The online version contains supplementary material available at 10.1186/s12864-023-09570-4.

## Introduction

The growth of plant organs directly depends on the parameters of the frequency of cell division, cell cycle and cell number and size, these processes are controlled by molecular mechanisms that coordinate and regulate cell cycle processes with nutrition, hormones, development, and environmental signals [1]. Molecular mechanisms are involved in the orderly progression of cells at various stages of the cell cycle and their appropriate response to extracellular cues [1]. Protein phosphorylation is well known as one of the major mechanisms that control cell cycle progression, it affects the cell cycle by altering protein activity, protein subcellular localization, target protein degradation, and dynamic changes in protein complex [2]. Among various kinases, cell cycle-dependent protein kinase (CDK) family is important for cell division control [3]. They form complexes with cyclin subunits and further drive the cell cycle by phosphorylating key target proteins needed for cells to move from one stage to the next [4]. The activity of CDK proteins is regulated in complex ways, such as through phosphorylation and/or dephosphorylation by specific kinases associated with regulatory proteins [5]. CDKs, a large class of serine/threonine protein kinases, were originally discovered due to their role in regulating the cell cycle, and they have different regulatory roles in many cell development processes in eukaryotes [6].

CDK proteins in plants can be divided into eight classes: CDKA to CDKG and cyclin-dependent kinase (CKL) [7]. CDK proteins of different subclasses in plant growth and development process have obvious structural and functional differences [8]. For example, Type A, which has a PSTAIRE motif and is responsible for binding to cyclin, the subfamily contains only one CDK gene, called CDC2A or CDKA, it was found in *Arabidopsis thaliana* Homozygous CDKA mutants can be distinguished by lack of root growth, significantly reduced cotyledon expansion and hypocotyl elongation, minute rosette leaf, and formation of fully sterile flowers [9]. There are two genes homologous to the CDKA gene in alfalfa in alfalfa: CDKA; 1; 1 and CDKA; 2; 1; they show multiple abilities of the complement yeast CDC28 mutant, whose cell cycle is blocked at the G1-S and G2-M phases, suggesting a distinct role for the CDKA gene in cell cycle regulation [10]. Type B CDKB with PPTALRE or PPTTLRE motifs, are plant-specific kinases that are involved in different cellular functions, and their expression is subject to strict cell cycle control [1]; ATCDKB1; 1 is highly expressed in guard cells and stomatal precursor cells of cotyledons, indicating that B-type CDK plays \ important roles in stomatal development [11]. The C-type CDKC kinases with PITAIRE motifs, are the closest homologs of the metazoan CDK9 protein, which has no defined role in cell cycle control and can form complexes with CYCT, it is hypothesized that its CTD by phosphorylating RNA polymerase II plays a role in transcriptional elongation [12, 13] and that CDKC2 has a key role in regulating cell division and drought responses in Arabidopsis [14]. The E-type CDK motif with the SPTAIRE motif, it was first discovered in alfalfa [15], and CDKE is a homologue of mammalian CDK8 that interacts with cyclin C, and negatively affects transcription as part of RNA polymerase II holoenzyme [16]. ATCDKE1 has been found to play crucia roles in floral organ and cell expansion in leaves [15]. CDKD can form complex with CYCH, which can be phosphorylated by CDKF to exert CAK enzyme activity [17]. G-type CDK with PLTSLRE motifs, among them, CDKG1 regulates AS of splicing factor U2AF65A in vegetative tissues and CalS5 Mrna in pollen [18]. CKL-type with (V, I, L) (K, R) FMAREI motifs where motif changes of residues depend on the type of CKL, ranging from CKL1 to CKL15 [8].

At present, CDK gene family has been identified in many plants, including tobacco (*Nicotiana tabacum*) [19], tomato (*Lycopersicon esculentum*) [20], rice (Oryza sativa) [21], potato (*Solanum tuberosum*) [22], alfalfa (*Medicago sativa*) [23] and so on. It has been reported that the reduction of mesophyll cell division in wheat is positively correlated with the inhibition of CDKA1 activity [24]. In addition to the primary role of CDK genes in regulating cell cycle processing [25], many studies have linked CDK genes to drought and salt stress tolerance in plants. Abiotic stresses such as low temperature, drought, salt and heavy metals can hinder plant growth and development, thus significantly reducing plant productivity and yield [26]. CDK genes from Arabidopsis have been reported to be involved in the drought stress response by regulating blue light-mediated stomatal opening and controlling reactive oxygen species (ROS) homeostasis [27]. In addition, the CDKG2 gene has been found to confer salt tolerance and promote flowering in *Arabidopsis thaliana* [28]. In a recent study, Zhao [14] showed that CDK2 increases cell division and drought tolerance in *Arabidopsis thaliana* by regulating cycle genes and stomatal development-related genes. In summary, CDK genes play important roles in enhancing plant tolerance to abiotic stresses.

Quinoa (*Chenopodium quinoa* Willd) is a facultative halophyte, adapting not only to high soil salinity but also to other abiotic stresses such as drought, high temperature, ultraviolet radiation, and low temperature [29]. It is rich in a variety of nutrients needed by human beings, with a variety of nutritional health effects [30]. Quinoa is a new type of functional food with high nutritional value and has broad application prospects [31]. Although the role of multiple CDK gene families in some species has been demonstrated in the context of plant growth and abiotic stress tolerance [19–23], few studies on CDK genes have been reported in quinoa, considering the potential regulatory role of CDK protein in plant growth and development in response to environmental stress, it is necessary to carry out genome-wide analysis of this gene family in quinoa. Therefore, in this study, we identified the CDKs gene of quinoa, and comprehensively analyzed the basic information of quinoa CDKs protein, gene structure, chromosome distribution, gene duplication and so on. Real-time quantitative polymerase chain reaction to determine expression patterns under drought and salt stress. These results will be helpful to understand the mechanism of cell cycle regulation and the function of CDK gene in quinoa under drought and salt stress.

## Materials and methods

### Plant materials and treatments

The quinoa cultivar long quinoa L1 was selected from Gansu Academy of Agricultural Sciences. Quinoa seeds were disinfected in 75% ethanol for 30 s, rinsed with sterile water for 3 times, then disinfected in 10% sodium hypochlorite solution for 15 min, rinsed with sterile water for 5 times, dried them and sown on MS solid medium, the culture was carried out at 25 °C. The seedlings were cultured at 25 ° C for 16/8 hours until germination. The germinated seeds were planted in 1:1:1 pot containing sand, perlite, and peat (15 seedlings per pot with a diameter18 cm ), it was cultured in in a solar greenhouse (humidity 60–70%, light time 12 h, day/night temperature 28 °C/18°C). The seedlings were treated with 20% PEG stress and 100 mmol/L NaCl stress when they were 2 months old. (1) PEG stress: watering the seedlings from the roots with 20% PEG, 200 ml per pot; (2) salt treatment: 100 mmol/L NaCl 100 ml per pot; 0.5 g per pot at 0, 3, 6, 9, 12 and 24 h after PEG and salt treatment; Three biological replicates were performed at each time point, and leaves were collected provisionally and stored in liquid nitrogen and then stored in a refrigerator at -80 ° C for subsequent experiments.

### Search and identification of CDK gene members in quinoa

The genome, CDS sequence, amino acid sequence and GFF annotation files of quinoa were obtained by Phytomoze V12.1 database (https://phytozome.jgi.doe.gov/pz/portal.html). *Arabidopsis thaliana* CDK protein sequences as target sequences to predict the CDK candidate genes of quinoa in the quinoa genome [32]. The obtained quinoa CDK candidate genes were predicted and analyzed by Pfam (http://pfam.xfam.org/family) [33], NCBI-CDD(Conserved Domains Database)(https://www.ncbi.nlm.nih.gov/cdd/) [34], and SMART (http://smart.embl-heidelberg.de/) [35] online tools for protein conserved domains of the CDK family, resulting in 22 CDK gene members were identified in quinoa. **Basic Physicochemical Properties and Phylogenetic Tree Analysis of Proteins**.

The amino acid number, isoelectric point, molecular weight and hydrophobicity of 22 quinoa CDK proteins were analyzed by ExPASy (https://web.expasy.org/protparam/) [36] with the default parameters, and the subcellular localization was analyzed by Psort-Prediction (http://psort1.hgc.jp/form.html) [37]. The CDK protein sequences of *Arabidopsis thaliana* (29), Rice (20) and Quinoa (22) were Multiple sequence alignment by Clustal W method in MEGA11 [38] software, the maximum likelihood was used to construct a phylogenetic tree using the poisson model with pairwise deletion set to 1000 repeats and other parameters are default. The illustration of the evolutionary tree + was through Evolview (http://120.202.110.254:8280/evolview) [39].

### Analysis of Gene structure and conserved motifs

According to the GFF annotation file information of CDK family genes, GSDS 2.0 (Gene Structure Display Server 2.0) (http://gsds.cbi.pku.e-du.cn/) [40] was used to analyze the structural features of CqCDK genes, and analyze the conserved motifs of amino acids by MEME (Multiple Em for Motif Elicitation) online software (http://meme-suite.org/tools/meme) [41], and the number of motif search is set to 10, other parameters default.

### Analysis of chromosome localization and gene replication

At the same time, according to the gene annotation information, TBtools software [42] was used to draw quinoa CDK gene chromosome mapping. Duplicate gene pairs were analyzed by TBtools, and the identified duplicated gene pairs analysis was performed using the Ka/Ks function of TBtools.

### Secondary structure, upstream cis-regulatory element analysis and protein interaction network mapping

The secondary structure of amino acids was analyzed by on-line software (https://npsaprabi.ibcp.fr/cgibin/npsa_automat.pl?page=npsa_sopma.html) [43]. According to the GFF annotation files of quinoa, the 2000 bp of DNA sequence upstream of transcription start site of CDK family gene sequence was extracted by using the sequence extraction function of TBtools, then PlantCARE (http://bioinformatics.psb.ugent.be/webtools/plantcare/html/) [44] was used to search and analyze the cis-regulatory elements in these promoter regions. The CDK protein interaction network was constructed based on orthologous proteins from the model plant *Arabidopsis thaliana*, and the network diagram was drawn by String software (option value > 0.8) (http://Stringdb.org/) [45].

### Expression analysis, RNA extraction and real-time quantitative PCR of CDK family genes

The expression data of CDK genes in quinoa were obtained from the transcriptome data of different tissues and organs (No: PRJNA394651), as well as the above-ground tissues of quinoa seedlings under drought, high temperature, salt and low phosphorus (No: PRJNA306026) stress, the data were standardized by Log2 method, and the heat map was drawn by TBtools.

Total RNA of leaves (100 mg) was extracted using the AG RNAEX Pro Reagent (AG, Shanghai), and the synthesis of cDNA used the Superscript TM III reverse transcriptase kit (AG, Shanghai). Gene-specific amplified primers were designed on NCBI-Primer designing tool (https://www.ncbi.nlm.nih.gov/tools/primer-blast/). RT-qPCR analysis was performed with 2 ×quantitect-sybr-green-pcr-mix (Qiagen) in the abi-viia 7 real-time PCR system of Applied Biosystems, USA, as follows: denaturation at 95 ° C for 30 S followed by denaturation at 95 ° C for 5 s for 40 cycles; At last, annealing/extension was carried out at 60 °C for 1 min. The experiment was repeated three times with an independent RNA sample.

### Subcellular localization of the protein encoded by *CqCDK15* gene

According to the principle of homologous recombination, the cloned *CqCDK15* gene was ligated into the expression vector containing green fluorescent protein (GFP), and the constructed vector plasmid was transformed into *Agrobacterium tumefaciens* strain GV1300 by electroporation, the PCAMBIA1302: : CqCDK15-GFP vector was constructed, and the vector was transferred into the protoplasts by the protoplast transient transfection technique [46], incubated for 16 h at shading room temperature, and a small amount of the protoplasts solution was dripped onto the slides with a pipette, gently cover the coverslip onto the slide at one end to avoid creating bubbles. A small amount of lens oil was added to the surface of the coverslips. Finally, the spatial expression of the protein encoded by *CqCDK15* was observed under a confocal laser microscope and recorded.

### Transformation of *Arabidopsis thaliana* with *CqCDK15* gene and determination of related physiological indexes

The coding sequence of *CqCDK15* gene (without the terminator sequence) was inserted into the pCAMBIA-1302-EGFP vector to obtain the recombinant plasmid. The recombinant plasmid pCAMBIA-1302: CqCDK15 was transferred into *Agrobacterium-tumefaciens* GV3101. After screening and identification of the positive clones, the transgenic Arabidopsis lines were obtained by infusing the inflorescences of *Arabidopsis thaliana* with floral dip method [47]. After the seeds of T0 generation were collected, the transgenic seeds of T0 generation were sterilized in 6.25% NaClO solution for 15 min, washed with ddH_2_O for 5 times, the positive plants were selected on MS medium containing 50 µg/ml hygromycin b until the homozygous transgenic plants of T3 generation were selected for further analysis.

PCR detection: the kanamycins resistant transgenic plants and the wild type *Arabidopsis thaliana* leaves were selected as materials, and their DNA was extracted respectively. The positive control was the recombinant plasmid DNA of CqCDK15-OE, wild-type DNA was the negative control. Reference primer pairs hyg-417 BP (F: AAATCCGCGTGCACGAGGT; R: TCGTTATGTTTATCGGCACTTTGCA); transgenic plant PCR detection was performed. For salt stress and drought treatment, seeds of transgenic and wild type lines were sterilized and cultured with 20% PEG and 100 mM NaCl, respectively. After growing on MS Medium at 22 ° C for 7 days, the plant phenotype was observed and the root length was measured.

### Statistical analysis

Each experiment was repeated at least three times and values were expressed as means ± SE. All the statistical analyses were performed using SPSS software (IBM, Armonk, NY) and OriginPRO8 software (OriginLab Corp., Northampton, MA, USA). Differences among means for treatments or lines were evaluated by Tukey HSD test at 0.05 levels. For assays the different measurements were subjected to a one-way analysis of variance (ANOVA).

## Results

### Basic physicochemical properties and phylogenetic tree analysis

Using the known CDK protein sequence of Arabidopsis, the whole genome of quinoa was compared and analyzed, and 22 CqCDK genes were identified. The Expasy was used to further confirm the 22 CDKs obtained by identification, and finally named as *CqCDK01*-*CqCDK22* (Table 1). The number of amino acid residues of CqCDK protein ranged from 173 aa (CqCDK15) to 811 aa (CqCDK04), and their molecular weight ranged from 19554.89 Da (CqCDK15) to 91375.70 Da (CqCDK04). Theoretical isoelectric points are located between 4.57 (CqCDK16) and 9.77 (CqCDK07), which can be roughly divided into two categories: basic proteins (CqCDK03-CqCDK09, CqCDK11-CqCDK15, CqCDK18, CqCDK20, CqCDK22) and acidic proteins (CqCDK01, CqCDK02, CqCDK10, CqCDK16, CqCDK17, CqCDK19, CqCDK21). The hydrophobicity index was less than 0, indicating that the proteins encoded by CDK genes were hydrophilic proteins. Subcellular prediction showed that eight CqCDK proteins were located in the nucleus, six in the microbody (peroxisome), six in the cytoplasm, one in the plasma membrane, and one in the mitochondria.

**Table 1:** Analysis of physicochemical properties of proteins encoded by CDK gene family

Gene accession No	Gene	Size (aa)	Molecular weight (D)	Isoelectric point	GRAVY	Subcellular Localization
AUR62024153RA	*CqCDK01*	755	85557.31	5.71	-0.881	Nucleus
AUR62029661RA	*CqCDK02*	591	66172.42	5.02	-0.519	Mitochondrial
AUR62002003RA	*CqCDK03*	506	57034.03	9.1	-0.606	Microbody
AUR62039460RA	*CqCDK04*	811	91375.70	7.06	-0.105	Microbody
AUR62003799RA	*CqCDK05*	506	57005.04	9.07	-0.601	Microbody
AUR62006460RA	*CqCDK06*	309	35055.56	8.26	-0.280	Cytoplasm
AUR62000247RA	*CqCDK07*	288	31905.51	9.77	-1.168	Nucleus
AUR62000549RA	*CqCDK08*	458	51581.29	9.34	-0.474	Nucleus
AUR62000572RA	*CqCDK09*	283	32415.87	7.07	-0.023	Microbody
AUR62020476RA	*CqCDK10*	636	72430.27	6.72	-0.633	Nucleus
AUR62006592RA	*CqCDK11*	327	36429.91	9.66	-0.968	Nucleus
AUR62006902RA	*CqCDK12*	472	53033.88	8.82	-0.375	Microbody
AUR62006925RA	*CqCDK13*	508	57247.82	8.47	-0.327	Nucleus
AUR62028402RA	*CqCDK14*	512	56898.13	9.26	-0.825	Nucleus
AUR62033763RA	*CqCDK15*	173	19554.89	9.49	-0.176	Cytoplasm
AUR62010957RA	*CqCDK16*	479	53527.81	4.57	-0.446	Cytoplasm
AUR62025896RA	*CqCDK17*	600	67472.56	5.14	-0.389	Plasma membrane
AUR62029816RA	*CqCDK18*	309	35015.49	8.26	-0.266	Cytoplasm
AUR62021462RA	*CqCDK19*	479	53441.72	4.58	-0.435	Cytoplasm
AUR62015735RA	*CqCDK20*	419	47562.72	9.59	-0.500	Cytoplasm
AUR62005577RA	*CqCDK21*	779	87463.3	5.84	-0.862	Nucleus
AUR62029943RA	*CqCDK22*	420	47159.09	8.99	-0.397	Microbody

**Fig. 1:**
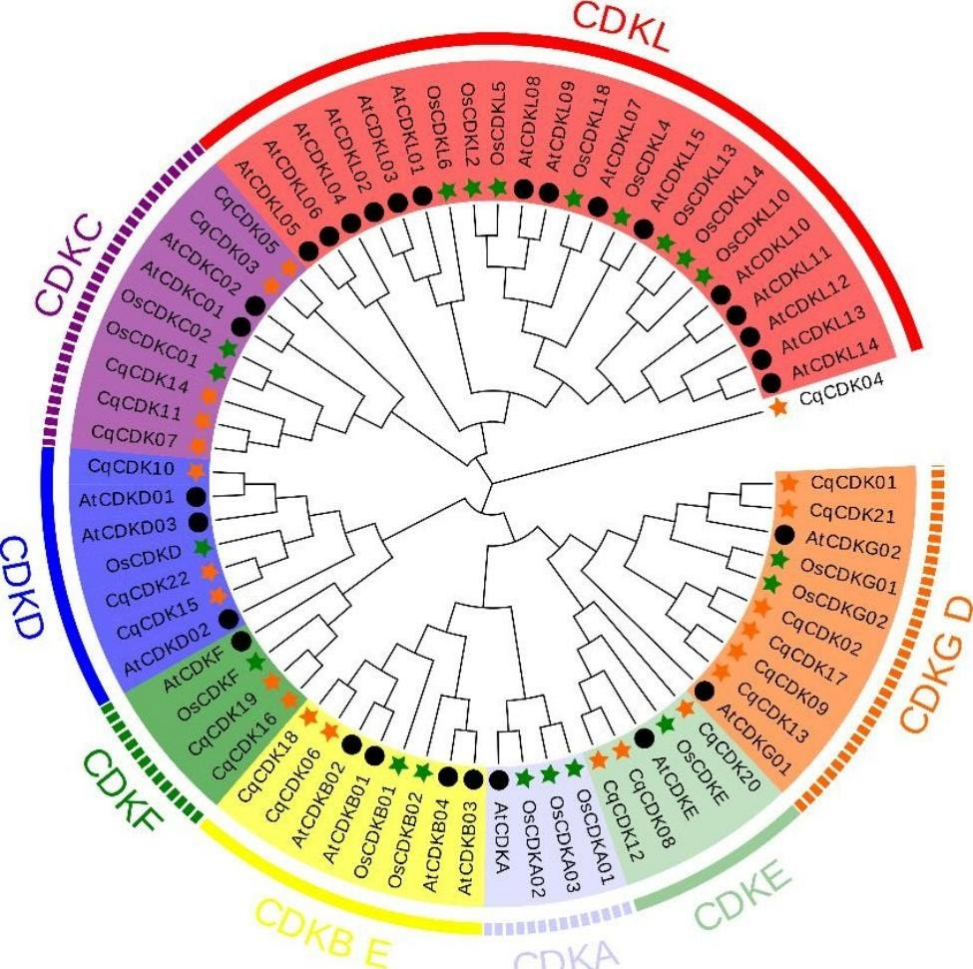
Phylogenetic tree of CDK gene in quinoa, Arabidopsis and rice Note: The phylogenetic tree was constructed using MEGA-11 based on the maximum natural method; bootstrap repeats 1000 times. Different subpopulations are highlighted in different colors. Black solid circle, orange solid star and green solid star represent CDK protein from *Arabidopsis thaliana*, quinoa and rice, respectively.

To further understand the CDK genes in quinoa, phylogenetic trees were constructed using 29 and 20 CDK genes from Arabidopsis and rice, respectively (Fig. 1, Table S1). The results showed that 21 CDK genes of quinoa were divided into six subfamilies, and *CqCDK04* gene showed a single branch. CDKA subfamily did not contain CDK genes of quinoa. CDKB subfamily contained two CqCDK genes (*CqCDK06* and *CqCDK18*), and CDKC subfamily contained five CqCDK genes (*CqCDK03*, *CqCDK05*, *CqCDK07*, *CqCDK11* and *CqCDK14*), CDKD subfamily contains three CqCDK genes (*CqCDK10*, *CqCDK15*, *CqCDK22*), CDKE subfamily contains three CqCDK genes (*CqCDK08*, *CqCDK12*, *CqCDK20*), and CDKF subfamily contains two CqCDK genes (*CqCDK09* and *CqCDK16*), CDKG subfamily contains four CqCDK genes (*CqCDK02*, *CqCDK09*, *CqCDK13* and *CqCDK17*). In phylogenetic tree analysis, the alleles of most genes are closely related. In quinoa, there were nine pairs of paralogous gene (*CqCDK01/CqCDK21*, *CqCDK02/CqCDK17*, *CqCDK09/CqCDK13*, *CqCDK08/CqCDK12*, *CqCDK06/CqCDK18*, *CqCDK16/CqCDK19*, *CqCDK07/CqCDK11*, *CqCDK03/CqCDK05*), no homologous genes were found in the CDK genes of quinoa, rice and Arabidopsis.

### Gene structure and basic motif analysis

**Fig. 2:**
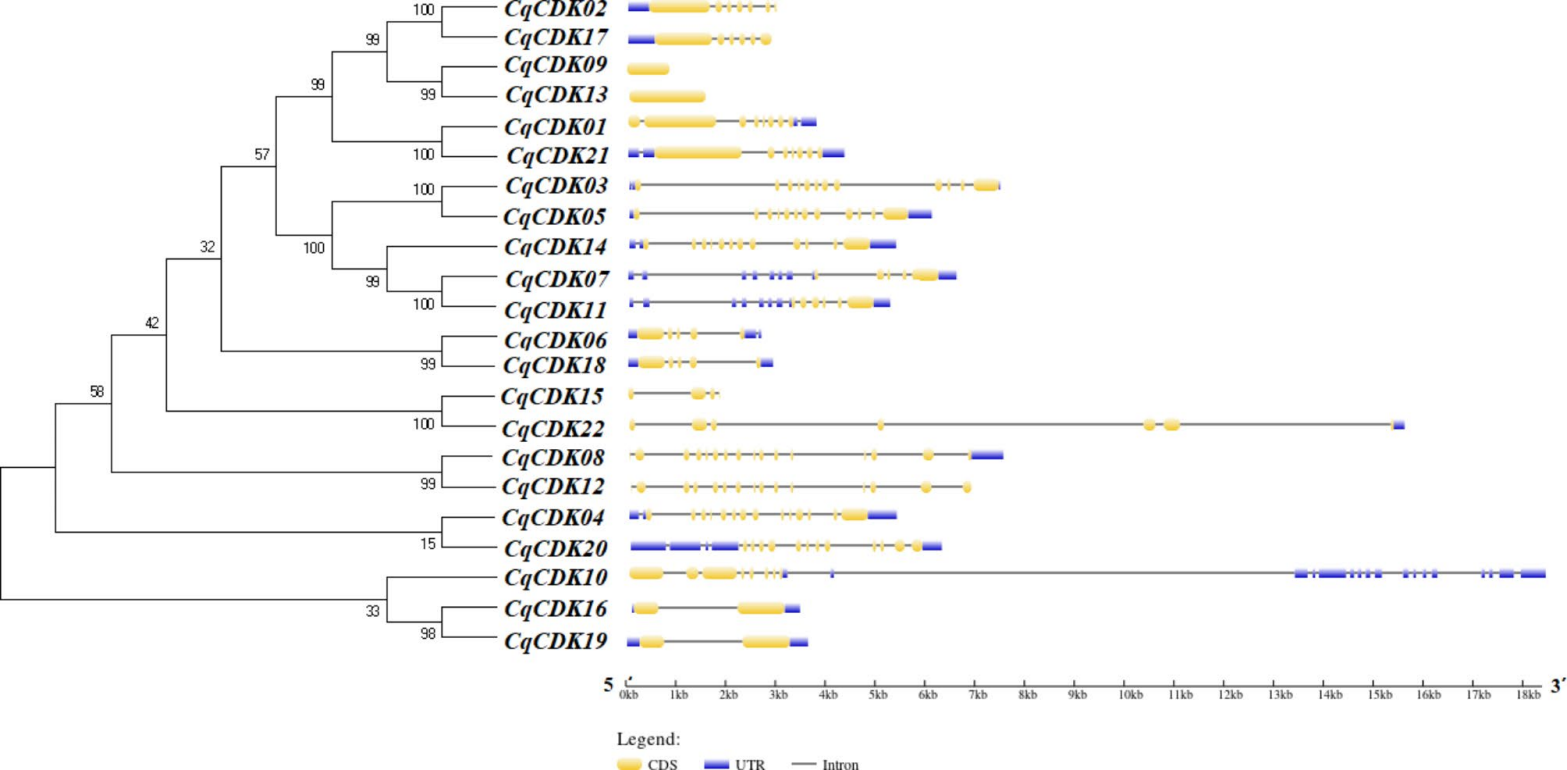
Gene structure of CDK gene in quinoa. Blue boxes indicate 5’-and 3’-untranslated regions; light yellow boxes indicate exons; and black lines indicate introns

The gene structure of exons/introns plays key roles in the evolution of gene families, so we analyzed the number and location of exons/introns in the CqCDK family, the results of the gene structure map (Fig. 2) showed that 12 CqCDK (54.5%) genes have UTR regions at 5’ and 3’ ends, and six CqCDK (27.3%) genes have UTR regions at only one end. *CqCDK09*, *CqCDK12*, *CqCDK13* and *CqCDK15* genes did not have UTR regions. *CqCDK08* has 16 exons, *CqCDK12* has 15 exons, *CqCDK04* has 14 exons, *CqCDK03*, *CqCDK05*, *CqCDK14* and *CqCDK20* have 12 exons, *CqCDK01* and *CqCDK10* have eight exons, *CqCDK02*, *CqCDK21* and *CqCDK22* have seven exons, *CqCDK11* and *CqCDK17* have six exons, *CqCDK06*, *CqCDK07* and *CqCDK18* have five exons, *CqCDK15* have four exons, and *CqCDK16* and *CqCDK19* have two exons, *CqCDK09* and *CqCDK13* have one exon.

**Fig. 3:**
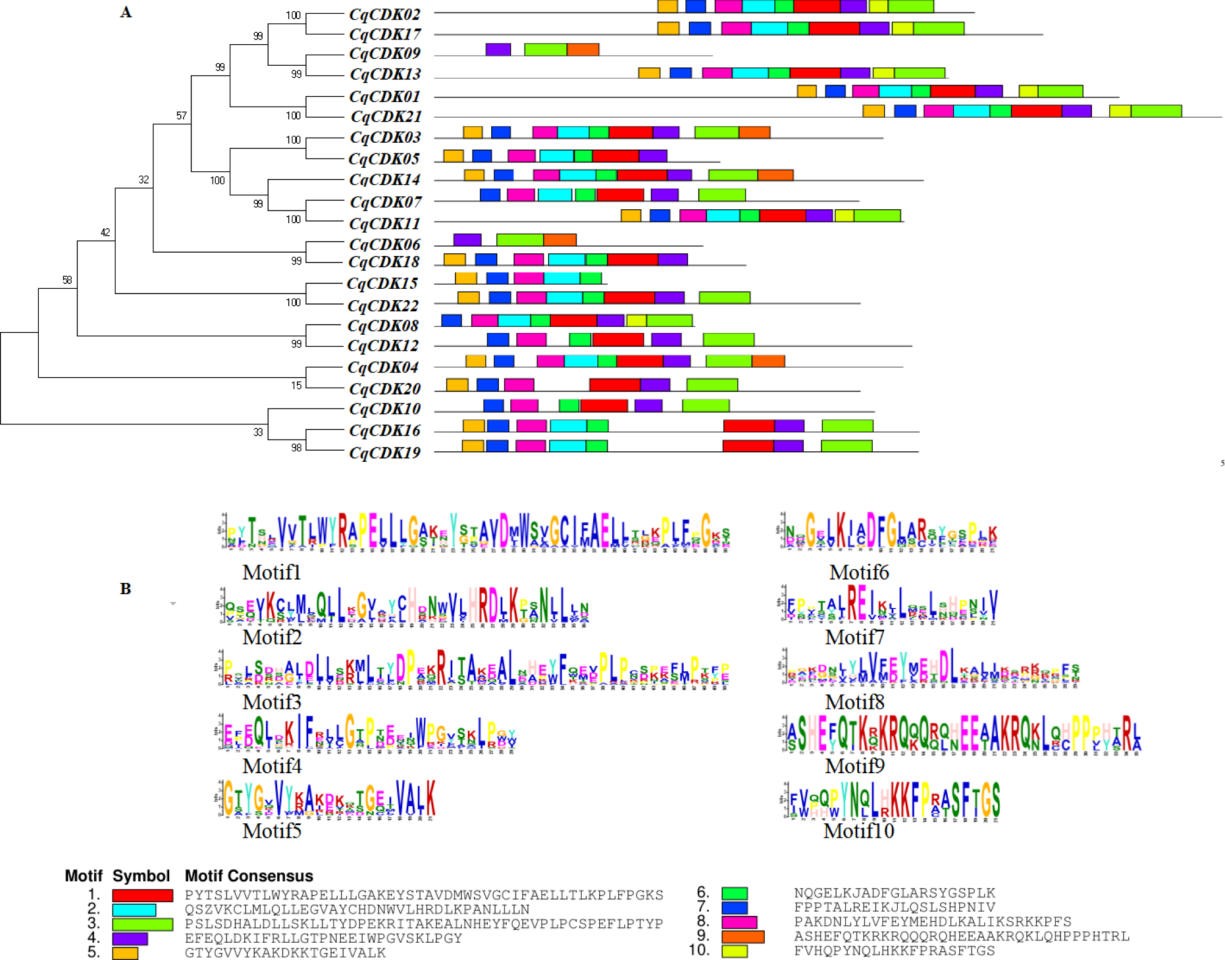
Conserved motif composition of CDK gene in quinoa. (**A**) The motif composition of CqCDK gene. (**B**) The conservative degree of the motif. (**C**) 10 conservative motif categories

The basic motifs of CDK family proteins were predicted by MEME software, and 10 conserved motifs were retrieved from the 22 CDK proteins in quinoa (shown in Fig. 3). We can observe that each CDK protein has different motifs (3–9 motifs), but genes with the same motifs are located in the same branch (as shown in Fig. 3 below), and motif 4 is present in 21 CqCDKs; motif 7 and Motif 8 are present in 20 CqCDKs, motif 1, motif 3, and motif 6 in 19 CqCDKs, motif 2 in 17 CqCDKs, and motif 5 in 16 CqCDKs, these results suggested that these eight motifs have conserved positions and functional similarities in quinoa. Meanwhile, individual motifs exist only in individual CqCDK proteins, Motif 9 was found in CqCDK03, CqCDK04, CqCDK06, CqCDK09 and CqCDK14, and Motif 10 was found in CqCDK01, CqCDK02, CqCDK08, CqCDK11, CqCDK13, CqCDK17 and CqCDK21, 40.9% of the genes contained 9 motifs (CqCDK02, CqCDK17, CqCDK13, CqCDK01, CqCDK21, CqCDK03, CqCDK11, CqCDK04 and CqCDK14), and 18.1% of the proteins contained 8 motifs (CqCDK22, CqCDK08, CqCDK16 and CqCDK19), 13.6% of the proteins contained seven motifs (CqCDK05, CqCDK18 and CqCDK07), which may be the basis for their classification. These motifs may have important functions in the quinoa CDK genes.

### Chromosome location and gene duplication analysis

**Fig. 4:**
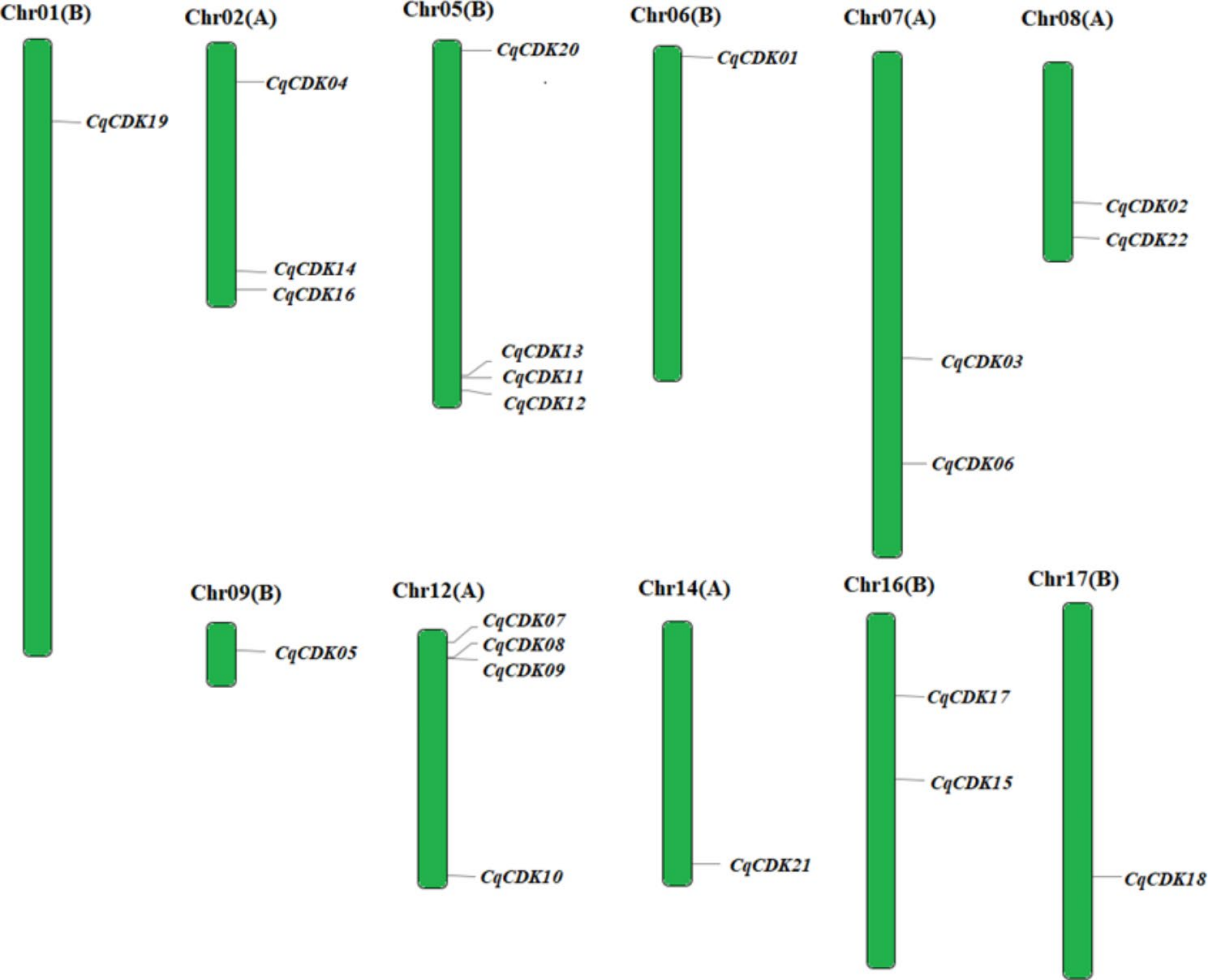
Genomic distributions of CDK genes on 11 chromosomes

To verify the relationship between genetic differentiation and gene duplication, we mapped the CqCDK genes obtained from quinoa database. The results showed that 22 CqCDK genes were evenly distributed on all 11 chromosomes of quinoa (Fig. 4), there are one CqCDK gene on chromosome Chr01(B), Chr06(B), Chr09(B), Chr14(A) and Chr17(B) respectively, two CqCDK genes on chromosome Chr07(A) and Chr16(B) respectively, and three CqCDK genes on chromosome Chr02(A), there are four CqCDK genes on Chr05(B) and Chr 12(A) chromosome.

We also analyzed the duplication of the quinoa CDK genes, and found 11 pairs of duplicated genes in the quinoa CDK gene through the relationship of high protein sequence homology and similarity, the sequence homology of amino acid composition was more than 75%. We can explore the potential functional differentiation and selection pressure of orthologs by calculating the ratio of Ka (non-synonymous) and Ks (synonymous) substitution rates. As shown in the table below, the Ka/Ks ratios of the allodiploids in this study were all less than1, indicating that the duplicated genes underwent purifying selection pressure (Table 2). The Ka value of duplicated gene (*CqCDK08/CqCDK12*) was higher than that of other homologs in the genome, indicating that the protein of this group of homologs evolved more rapidly. (*CqCDK09/CqCDK13*) had the highest Ka/Ks ratio (0.446), thus, this pair of gene experienced higher evolutionary pressure in quinoa compared to other genomes. The vast majority of gene duplication occur within ten million years. The first group of duplicated gene (*CqCDK09/CqCDK02*) occurred 45.124 million years ago, the last group of genes to be homologous (*CqCDK07/CqCDK11*) occurred 3.385 million years ago.

**Table 2:** Gene duplication analysis of CDK gene family in quinoa

Duplicated SnRK2 gene1	Duplicated SnRK2 gene2	Ka	Ks	Ka/Ks	MYA	Selective pressure	Duplicate type
CqCDK01	CqCDK21	0.016	0.115	0.141	7.653	Purifying selection	Segmental
CqCDK02	CqCDK17	0.025	0.170	0.145	11.322	Purifying selection	Segmental
CqCDK03	CqCDK05	0.020	0.089	0.221	5.901	Purifying selection	Segmental
CqCDK06	CqCDK18	0.006	0.133	0.043	8.887	Purifying selection	Segmental
CqCDK07	CqCDK11	0.012	0.051	0.242	3.385	Purifying selection	Segmental
CqCDK08	CqCDK12	0.050	0.130	0.387	8.637	Purifying selection	Segmental
CqCDK09	CqCDK13	0.048	0.109	0.446	7.244	Purifying selection	Segmental
CqCDK15	CqCDK22	0.021	0.076	0.280	5.098	Purifying selection	Segmental
CqCDK16	CqCDK19	0.007	0.060	0.121	4.004	Purifying selection	Segmental
CqCDK09	CqCDK17	0.127	0.651	0.195	43.402	Purifying selection	Segmental
CqCDK09	CqCDK02	0.122	0.677	0.180	45.124	Purifying selection	Segmental

### CDK Gene Family coding protein secondary structure analysis

The secondary structure showed that the proteins encoded by CqCDK family all contained alpha helix, extended strand and random coil. The α-helix structure of *CqCDK15* gene was the highest (45.66%), which was composed of 79 amino acids (Table S2). The random coil structure was the highest in the remaining CDK genes. The α-helix structure of *CqCDK02*, *CqCDK09* and *CqCDK13* genes was the lowest, and the derivative chain structure of other CDK genes was the lowest, among which 17 genes accounted for less than 20%. In CqCDKs, the proportion of random coil structure in some gene-encoded proteins was more than 50%.

### Cis-regulatory element analysis and protein Interaction Network Construction

**Fig. 5:**
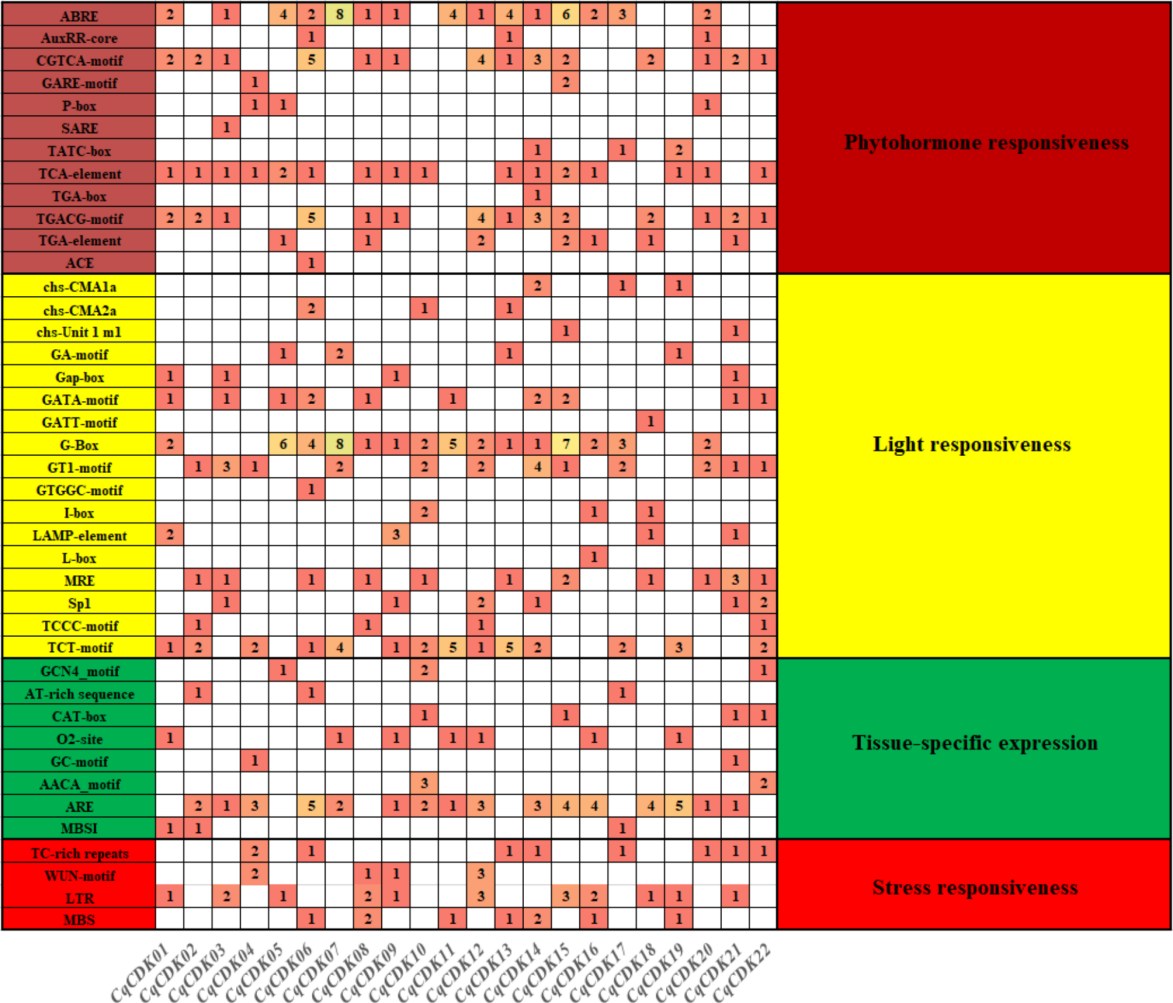
Cis-elements in the promoter regions of CDK genes in quinoa

To investigate the cis-regulatory element of the promoter region of the CqCDK gene, we analyzed the 2000 bp sequence upstream of the transcription start site with PlantCARE. A total of 41 cis-regulatory element related to plant hormone responses, the abiotic stresses, tissue-specific expression, and photoresponse were identified (Fig. 5). At the same time, we observed that different genes had different types and different amounts of cis-regulatory element. Among them, GATA-motif, G-Box, GT1-motif, LAMP-element, MRE, SP1 and TCT-motif are the most common cis-regulatory elements, AUXRR-core only existed in *CqCDK06*, *CqCDK13* and *CqCDK20* genes, GARE-motif only existed in *CqCDK04* and *CqCDK14* genes, P-box only existed in *CqCDK04*, *CqCDK05* and *CqCDK20* genes, and SARE only existed in *CqCDK03* gene, TAC-box only existed in *CqCDK14*, *CqCDK17* and *CqCDK19* genes, TGA-box only existed in *CqCDK14* gene, ACE only existed in *CqCDK06* gene. The second is tissue-specific expression element, in which the cis-regulatory element (ARE) necessary for anaerobic induction exists in multiple copies of CqCDK genes, GCN4-motif exists in *CqCDK05*, *CqCDK10* and *CqCDK22* genes, AT-rich sequence only exists in *CqCDK02*, *CqCDK06* and *CqCDK17* genes, GC-motif only exists in *CqCDK04* and *CqCDK21* genes, AACA-motif only exists in *CqCDK10* and *CqCDK22* genes, MBSI only exists in *CqCDK01*, *CqCDK02* and *CqCDK17* genes. In terms of plant hormone response elements, in which ABRE, CGTCA-motif, TCA-elements, TGACG-motif exists in multiple copies of CqCDK genes, and *CqCDK06*, *CqCDK14*, *CqCDK15* and *CqCDK20* genes contain six hormone response elements, including abscisic acid, methyl jasmonate, gibberellin, salicylic acid and auxin, *CqCDK07* only contains abscisic acid acting element, *CqCDK10* only contains salicylic acid acting element, and *CqCDK11* only contains abscisic acid acting element, *CqCDK17* only contains gibberellin-acting element and abscisic acid-acting element, *CqCDK19* only contains salicylic acid-acting element and gibberellin-acting element. These results suggested that different CqCDK genes play specific roles in plant growth and development by responding to different hormone signals. Finally, there are stress-response elements, including cis-regulatory element (Tc-rich repeats), wound-response elements (WUN-motif), the cis-regulatory element (LTR) and drought-related functional elements (MBS) involved in the low temperature reaction. The cis-regulatory element involved in defense and stress responses, the cis-regulatory element involved in low temperature responses, and the elements involved in drought induction are present in many genes, the WUN-motif reaction element only exists in *CqCDK04*, *CqCDK08*, *CqCDK09* and *CqCDK12* genes.

**Fig. 6:**
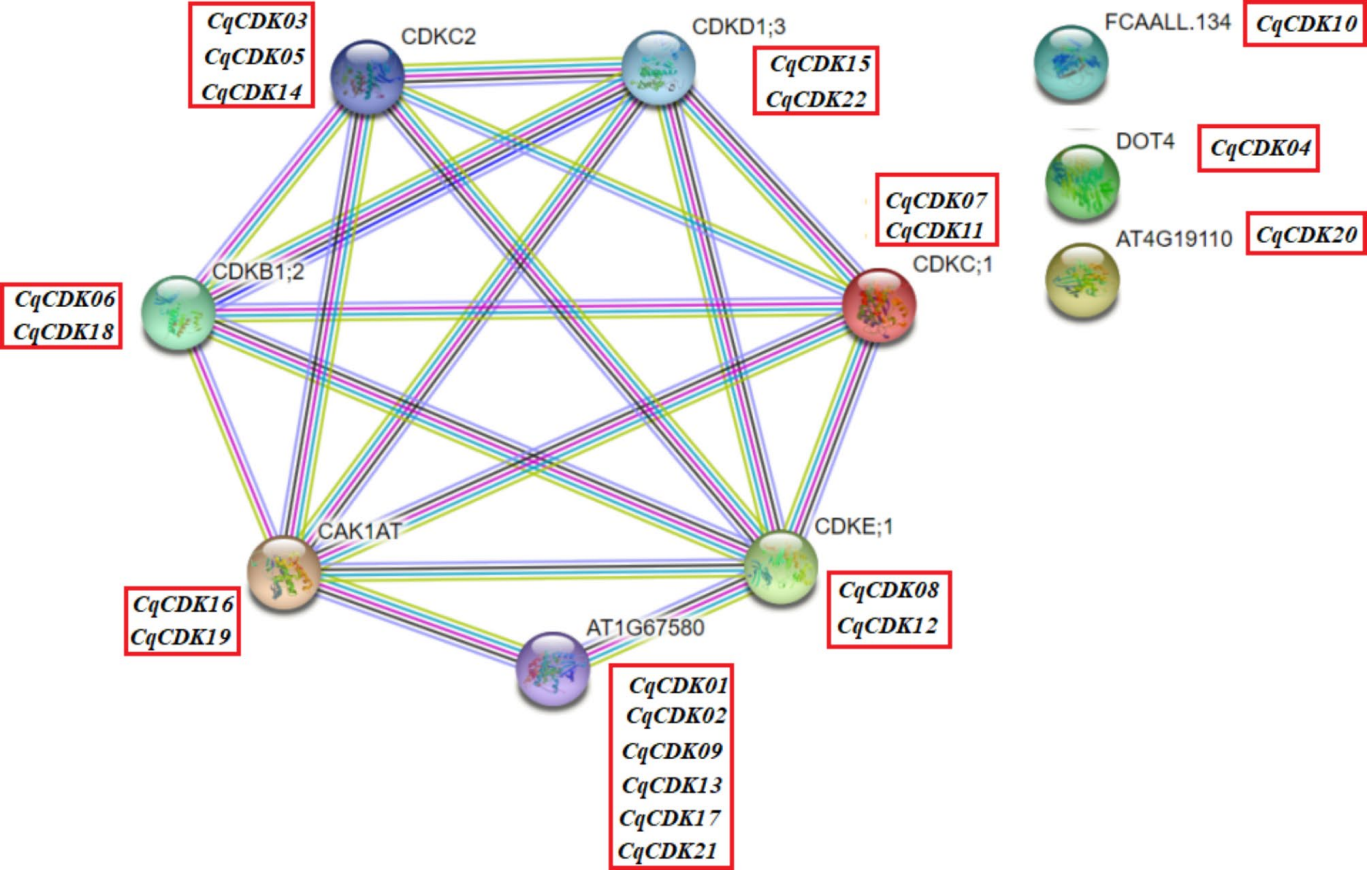
Analysis of CDK protein network interaction in quinoa

To predict the interaction and association between all CqCDK proteins, we constructed a CDK protein interaction network diagram between *Arabidopsis thaliana* and quinoa using String software, and based on the predicted results, all the CqCDK-like proteins appear in the known interaction network of Arabidopsis CDK proteins (Fig. 6), indicating that there is a complex relationship between the two. We can observe that the protein sequence of AtCDKC2 is highly similar to that sequence of three CqCDK proteins (CqCDK03, CqCDK05 and CqCDK14), AtCDKD1; 3 is highly similar to that of two CqCDK proteins (CqCDK15 and CqCDK22), The protein sequences of AtCDKC;1 were highly similar to those of the two CqCDK proteins (CqCDK07 versus CqCDK11) and the protein sequences of AtCDKE;1 were highly similar to those of the two CqCDK proteins (CqCDK08 versus CqCDK12) ; The protein sequence of AT1G67580 was highly similar to that of six CqCDK proteins (CqCDK01, CqCDK02, CqCDK09, CqCDK13, CqCDK17 and CqCDK21). The protein sequence of CAK1AT was highly similar to that of two CqCDK proteins (CqCDK16 and CqCDK19). The protein sequence of FCAALL.134 was highly similar to that of one CqCDK protein (CqCDK10), and the protein sequence of FCAALL.134 was highly similar to that of two CqCDK proteins (CqCDK06 and CqCDK18), the protein sequence of Dot4 is highly similar to CqCDK04 protein sequence, and the protein sequence of AT4G19110 is highly similar to CqCDK20.

*CqCDK09* gene and *CqCDK13* gene were expressed in different degree under different stress (Table S3), and the expression levels of *CqCDK09* gene and *CqCDK13* gene were lower under eight stress treatments, the expression levels of *CqCDK10* and *CqCDK20* genes were higher under eight stress treatments (Figure S1). Under Root-dry treatment, two genes (*CqCDK02* and *CqCDK15*) showed significantly up-regulated expression and three genes (*CqCDK06*, *CqCDK14* and *CqCDK19*) showed significantly down-regulated expression after drought treatment. Under Root-heat treatment, the expression level of eight genes (*CqCDK03*, *CqCDK05*, *CqCDK08*, *CqCDK11*, *CqCDK13*, *CqCDK16*, *CqCDK19* and *CqCDK22*) was down-regulated, while the expression level of *CqCDK02* gene was up-regulated. Under Root-Low-P treatment, the expression level of 12 genes (*CqCDK01*, *CqCDK03*, *CqCDK05*, *CqCDK06*, *CqCDK08*, *CqCDK12*, *CqCDK14*, *CqCDK16*, *CqCDK18*, *CqCDK19*, *CqCDK21* and *CqCDK22*) was down-regulated, while the expression level of *CqCDK02* was up-regulated. Under Root-salt treatment, the expression level of four genes (*CqCDK03*, *CqCDK06*, *CqCDK14* and *CqCDK18*) was down-regulated, while the expression level of *CqCDK09* was up-regulated. Under Shoot-dry treatment, the expression level of five genes (*CqCDK06*, *CqCDK07*, *CqCDK15*, *CqCDK18* and *CqCDK22*) was down-regulated, while the expression level of three genes (*CqCDK02*, *CqCDK13* and *CqCDK20*) was up-regulated. Under Shoot-heat treatment, the expression level of three genes (*CqCDK06*, *CqCDK09*, *CqCDK22*) genes was down-regulated and three genes (*CqCDK02*, *CqCDK13*, *CqCDK17*) was up- regulated. Under Shoot-low-P treatment, the expression level of 13genes (*CqCDK01*, *CqCDK03, CqCDK05*, *CqCDK08*, *CqCDK09*, *CqCDK12*, *CqCDK14*, *CqCDK15*, *CqCDK16*, *CqCDK18*, *CqCDK19*, *CqCDK21* and *CqCDK22*) was down-regulated, while the expression level of three genes (*CqCDK02*, *CqCDK06* and *CqCDK17)* was up-regulated. Under Shoot-salt treatment, the expression level of eight genes (*CqCDK04*, *CqCDK06*, *CqCDK07*, *CqCDK09*, *CqCDK14*, *CqCDK17*, *CqCDK18* and *CqCDK22*) was down-regulated, while the expression level of two genes (*CqCDK13* and *CqCDK16*) was up-regulated.

### Expression pattern analysis under abiotic stress

**Fig. 7:**
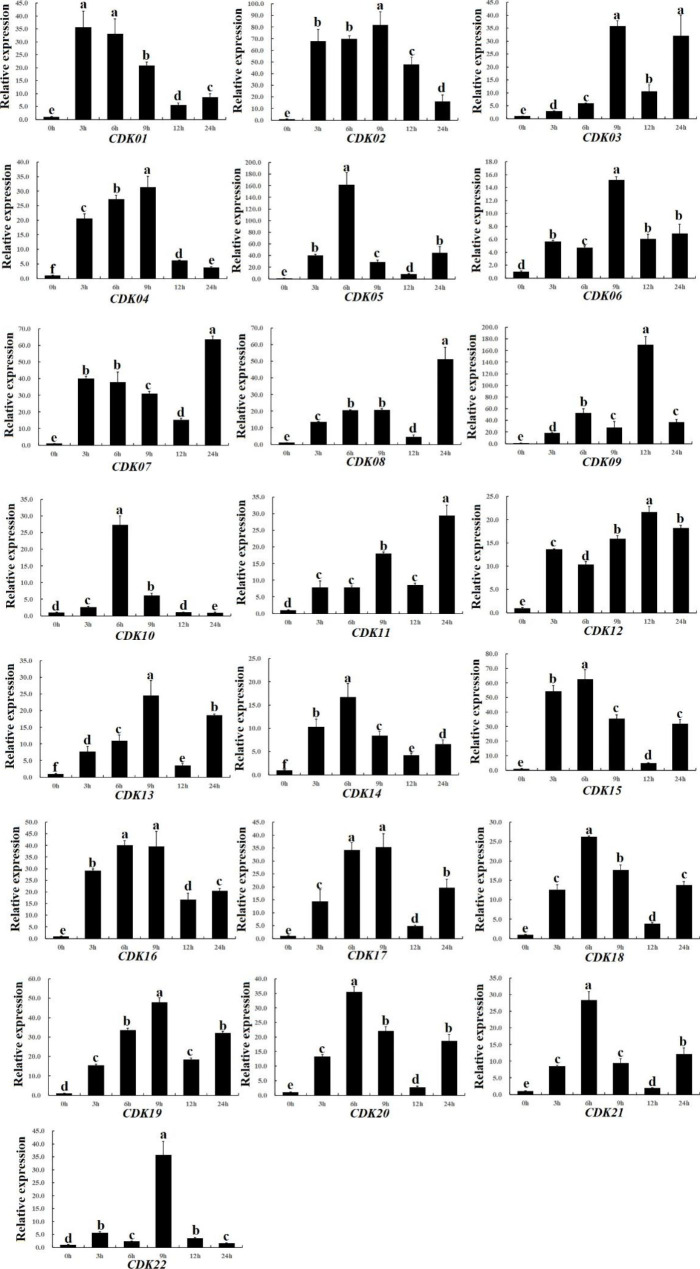
Expression pattern analysis under salt stress. qPCR was used to quantify the gene expression levels of 22 CDK genes from quinoa leaves under salt stress. Data are means ± SE of three independent biological samples, with vertical lines indicating standard deviation

CDK genes play key roles in abiotic stress responses, and therefore, we investigated the expression pattern of all CDK family genes in quinoa leaves under 100 mmol/L NaCl stress (Fig. 7, Table S4). The results showed that the expression level of *CqCDK05* and *CqCDK09* was up-regulated under salt stress, and the relative expression level of *CqCDK05* reached 162 at 6 h after salt stress. The expression level of *CqCDK02* gene was 81-fold higher than that of the control group. Except *CqCDK05* and *CqCDK09* genes, the expression level of remaining CDK genes under salt stress was below 100. Meanwhile, the expression level of some CDK genes was lower under salt stress. For example, *CqCDK06* gene reached its maximum value (15.17702), *CqCDK12* gene reached its maximum value (21.58169) and *CqCDK13* gene reached its maximum value (24.55857) after 9 h of salt stress, *CqCDK14* gene reached the maximum value (16.7331) at 6 h after salt stress, and the expression level was all lower than 25. In addition, the expression patterns of CqCDK genes under salt stress can be divided into the following categories: The first group, after 3,6 and 9 h of treatment, the expression level of CqCDK genes was significantly increased compared with the control, for example, *CqCDK01*, *CqCDK02*, *CqCDK04*, *CqCDK14*-*CqCDK20*. In the second group, the expression level of *CqCDK10* and *CqCDK21* genes reached the maximum after 6 h of processing. In the third group, the expression level of *CqCDK03*, *CqCDK05*, *CqCDK06*, *CqCDK09*, *CqCDK13* and *CqCDK22* reached the maximum value after 9 h of processing. In the fourth group, the expression level of *CqCDK07*, *CqCDK08* and *CqCDK11* genes reached the maximum after 12 h of processing. In addition, the expression level of some genes, such as *CqCDK12*, was significantly higher than that of the control at each time point after salt stress. These results suggested that the genes in this family are strongly responsive to salt stress.

**Fig. 8:**
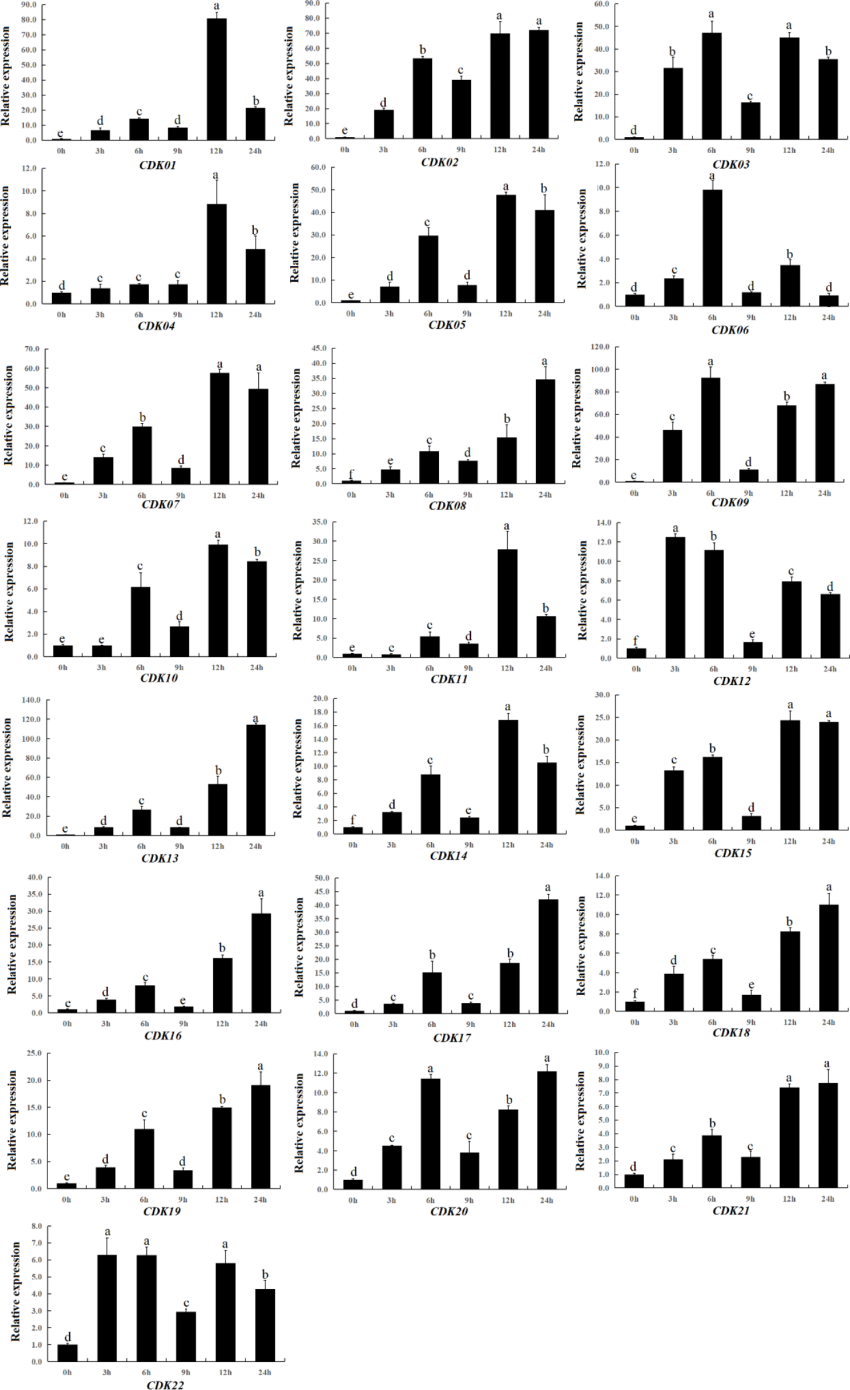
20% expression pattern analysis under PEG stress. qPCR was used to quantify the expression levels of 22 CDK genes from quinoa leaves under 20% PEG stress. Data are means ± SE of three independent biological samples, with vertical lines indicating standard deviation

In addition, we also studied the expression pattern of this family of genes under 20% PEG stress (Fig. 8). It was found that 22 CDK genes also strongly responded to drought stress. The expression level of *CqCDK13* was up-regulated to 100-fold under drought stress. The expression level of *CqCDK09* reached its peak at 6 h after drought stress (92.73395). The expression level of *CqCDK01* reached its peak at 12 h after drought stress (81.06545), and the expression of *CqCDK01* was up-regulated to 81-fold. The expression level of three genes (*CqCDK01*, *CqCDK09* and *CqCDK13*) was all significantly up-regulated under drought stress. At the same time, some of the CqCDK genes showed a relatively low expression level under drought stress. For example, *CqCDK04* gene reached its maximum value (8.846827) after 12 h of drought stress, the *CqCDK06* gene reached its maximum value (9.824998) at 6 h after drought stress, *CqCDK21* gene reached the maximum value (7.734927) and *CqCDK22* gene reached the maximum value (6.297493) after drought stress for 24 h, and their expression levels were all lower than 10. In addition, the expression patterns of CqCDK genes under drought stress can be broadly divided into the following categories: The first group, the expression level of *CqCDK12* and *CqCDK22* reached the maximum at 3 h after treatment. In the second group, the expression of *CqCDK03*, *CqCDK06* and *CqCDK09* genes reached the maximum at 6 h after treatment. In the third group, the expression of *CqCDK01*, *CqCDK02*, *CqCDK04*, *CqCDK05*, *CqCDK07*, *CqCDK10*, *CqCDK11*, *CqCDK14* and *CqCDK15* reached the maximum at 12 h after treatment. In the fourth group, the expression of *CqCDK08*, *CqCDK13*, *CqCDK16*-*CqCDK21* reached the maximum at 24 h after treatment. These results suggested that CqCDK genes are strongly responsive to drought stress.

### Subcellular localization analysis

**Fig. 9:**
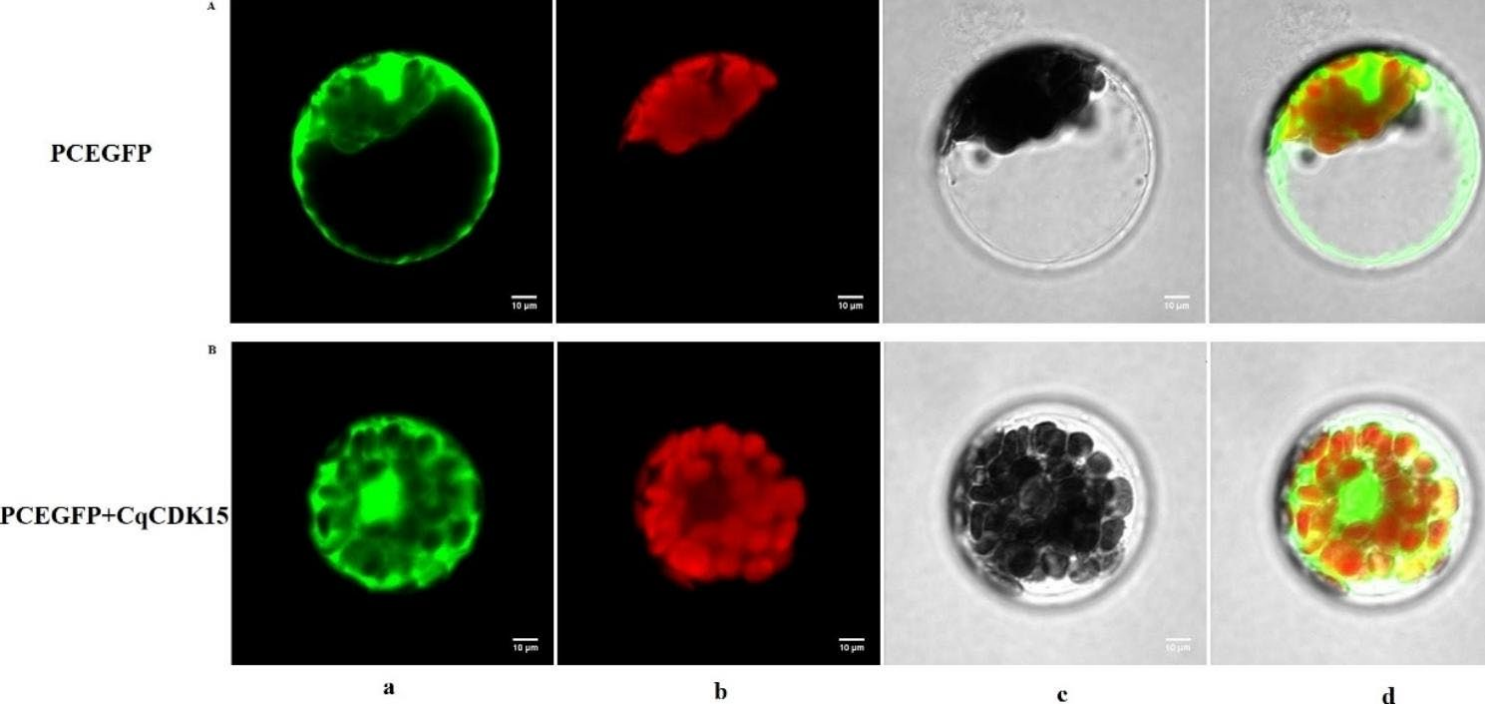
Confocal microscopy of (**A**) GFP, (**B**) CqCDK15-GFP localization. (**a**) Target protein fluorescence channel; (**b**) Chloroplast fluorescence channel; (**c**) Bright field; (**d**) Superposition field. Confocal micrographs showing CqCDK15 localization in mesophyll protoplasts validate the cytosol marker mkate (red)

To clarify the function of *CqCDK15*, we constructed the fusion expression vector of *CqCDK15* and green fluorescent protein, PCAMBIA1302::CqCDK15-GFP, and used the transient expression method of PEG4000-mediated transformation of *Arabidopsis thaliana* protoplasts, subcellular localization of *CqCDK15* was performed, with Empty-GFP, which was not linked to the target gene, as control, and the results showed that CqCDK15-GFP fusion protein was expressed in the nucleus and the cytoplasm (Fig. 9).

### Analysis of drought resistance and salt resistance in over-expressing *Arabidopsis thaliana*

**Fig. 10:**
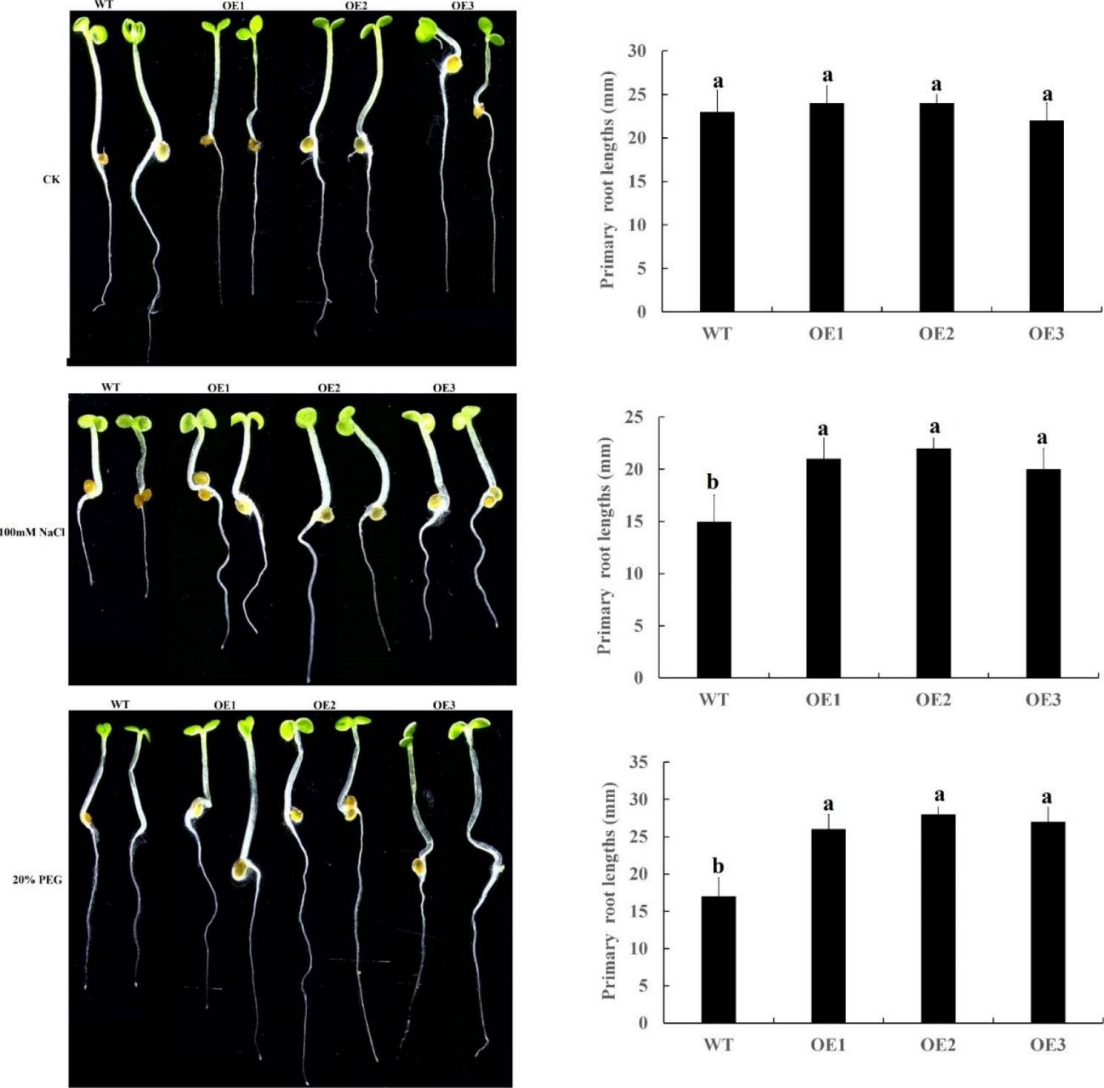
Effect of salt stress and drought stress on root length of wild-type (WT) and *CqCDK15* overexpressed (OE-1, OE-2, and OE-3) *Arabidopsis thaliana*. Different lowercase letters showed significant difference at 0.05 level (*P < 0.05*). These data are means ± SE of three independent biological samples, with vertical lines representing standard deviation

In order to further study the function of *CqCDK15* gene, *CqCDK15* was transferred into *Arabidopsis thaliana* by *Agrobacterium tumefaciens*-mediated transformation. After screening, T3 generation over-expression line (OE-1, OE-2, OE-3) were obtained, then it was treated with 20% PEG and 100 mm NaCl (Fig. 10). From the figure below we can see that there were no significant differences in root length between transgenic plants and WT plants without treatment. Under the stress of 100 mM NaCl and 20% PEG, we found that there were significant differences in root length between transgenic lines and wild-type lines. Under 100 mm NaCl stress, we found that the average root lengths of transgenic plants were significantly longer than that of WT plants, and the average root lengths in the transgenic plants were 1.4-fold that of wild-type plants. In the 20% PEG treatment, the average root lengths of transgenic plants were significantly longer than that of WT plants and 1.59-fold longer than that of WT plants. These results indicate that *CqCDK15* participates the regulation of drought and salt stress responses, and that over-expression of *CqCDK15* may make transgenic Arabidopsis plants more tolerant to drought and salt tolerance.

## Discussion

The function of the CDK genes has been extensively studied in many plant species. At present, there is no detailed analysis of the CDK genes in quinoa, so its function is still unknown. In this study, we identified 22 CDK genes from quinoa genome. A total of 57 CDK genes have been identified in the cotton. 31,15, and 12 CDK genes were identified in the G. hirsutum, G. raimondii and G. arboreum [48], while the number of genes identified in quinoa was relatively higher than that in G. Arboreum and G. Raimondii, which may be due to genome-wide duplication. In addition, studies have shown that there are 34 and 29 CDK genes in rice and Arabidopsis [7], respectively. Whereas 22 CDK genes were found in quinoa, and this difference may be related to genome size in different species. In addition, phylogenetic analysis showed that the six subfamilies contained CDK genes of Monocots and Dicotyledones. This analysis suggests that members of the CDK family are descendants of ancient copies that occurred before the Monocots and Dicotyledones split. As a result of subsequent duplication, the CDK gene is more abundant in the monocots. The high number of CDK genes during polyploidy is more likely to be caused by gene duplication and conservation of CDK genes during polyploidy, this suggests that the CDK gene family play important roles in plant growth and development [49].

Gene structure is usually conserved during evolution [50]. Gene structures such as intron/exon organization and intron type are typical imprints in the evolution of some gene families, the characterization and structural analysis of genes with major functions of abiotic and biological stress factors have been found to have fewer introns [51]. Structural analysis of the CDK gene in quinoa revealed changes in the intron-exon ratio, 12 CqCDK genes all have UTR regions at the 5 ‘and 3’ ends, and seven of them had exons with more than 10 exons. Two genes had no introns, and the rest were either destroyed by one or more introns, this may be due to the CDK genes in the evolution of intron loss, which is consistent with previous studies [52]. However, it is important to note that despite the burden of gene function on introns, CqCDK08 has the highest disruption, with an exon number of 16, due to the demand for spliceosomes. Studies have shown that evolutionarily conserved genes have a greater intron burden, and there is a positive correlation between evolutionarily conserved levels and the size of gene intron regions in eukaryotic genes [53], and the more introns there are, the more conserved the genes are [50], therefore CqCDK08 is the most conservative. Motif analysis and composition of each CDK gene subfamily varied widely, although some amino acid-rich regions were detected, which is consistent with findings in Arabidopsis [54] and tomato [55]. We found that the genes belonging to the same family showed similar gene structure and motif composition. This result is consistent with a previous study that documented similar exon, intron data and protein motifs in the same set of CDK genes in a genome-wide study in tomato [55]. Previous studies have shown that gene organization plays important roles in the evolution of multiple gene families and in response to stress conditions [56]. In our study, we found that the most closely related CqCDK genes in the same family share similar gene structures, this may indicate that gene structure reveals a phylogenetic relationship. Furthermore, cytoplasmic CqCDK genes typically have fewer introns, whereas organelle-specific CqCDKs have more introns, suggesting that intron patterns are closely related to gene function, and this diversity in gene structure may drive the evolution of gene families and may give genes new functions that help plants better adapt to environmental changes [57]. Furthermore, although all 21 CDK genes share a common conserved Motif4, they also have their unique conserved motifs, and the different motif composition may contribute to the functional diversity of CDK members [58].

Gene duplication plays important roles in the expansion and evolution of gene families [59]. In addition, gene duplication is also an important source of plant stress response mechanism innovation [60]. Gene duplication mechanisms not only lead to the expansion of genomic content, but also contribute to the diversification of gene function to ensure adequate fitness and plant evolution [61]. Therefore, we investigated gene duplication events between CDK genes in the quinoa genome to gain a deeper understanding of their evolutionary processes. The results showed that 22 CqCDK genes were derived from segmental duplication events, which indicated that segmental duplication was the main evolutionary force of CDK gene expansion, this is consistent with previous results obtained in cyclin-dependent kinase analysis in *Arabidopsis thaliana*, in which 22 core cell cycle genes were found to be duplicated by segmented repeats [62]. Furthermore, the Ka/Ks ratio of the CqCDK gene pair indicated that the replicated CqCDK gene pair was driven by purifying selection, with the Ka/Ks ratio < 1 [63], indicating that the replication event accelerated the generation of the CqCDK gene. Furthermore, these CqCDK gene replication events occurred between approximately 3.385 Mya and 45.124 Mya. Meanwhile, in paralog gene pairs, CqCDK07-CqCDK11 showed the lowest Ka/Ks values, suggesting that this pair of genes had a shorter divergence time, so these genes retained the same function.

Cis-regulatory elements, as key molecular switches, are involved in regulating the transcriptional regulation of gene activity in various biological processes [64]. In this study, we identified several hormones associated with auxin, gibberellin, salicylic acid (SA), abscisic acid (ABA) and methyl jasmonate (Meja) in the promoter region of the CqCDK cis-regulatory element. Abre (ABA response element) and G-BOX element were found in 15 CqCDK genes, TGACG-motif (MeJA response element) and ARE element were found in 15 CqCDK genes, this indicates that these components are highly conserved in the CqCDK family. Among them, ABRE plays an important role in response to ABA to affect osmotic stress and drought stress tolerance in plants [65, 66], the 15 CqCDK genes containing ABRE elements were also strongly responsive to drought stress. In addition, G-BOX is involved in the biosynthesis of linalool during flower development [67], suggesting that the CDK promoter region may be involved in the development of Quinoa. At the same time, the CDK gene contains two or more identical cis-regulatory element copies, which may play key roles in enhancing gene transcriptional regulation and adaptation to environmental changes. In addition, we found that the drought response element MBS existed in the promoter regions of *CqCDK06*, *CqCDK08*, *CqCDK11*, *CqCDK13*, *CqCDK14*, *CqCDK16* and *CqCDK19* genes. Meanwhile, it was observed that most of these genes responded strongly to drought stress 12 or 24 h after drought stress, suggesting that there was a close relationship between promoter elements and gene expression.

The results showed that most of the CqCDK genes were significantly induced under drought and salt stress, and the expression of most of the CqCDK genes remained at a high level, which was similar to the results of other early studies, these results suggest that CDK plays important roles in the stress response of quinoa to drought stress. Therefore, the aim of this study was to further understand the expression pattern and possible function of the CqCDK gene under drought and salt stress. Almost all of the 22 CqCDK genes were induced by drought and salt stress. These results suggest that the accumulation of CqCDK genes are effective in reducing abiotic stress-induced damage. CDKC2 regulates cell division and drought stress in *Arabidopsis thaliana* by mediating cell cycle genes and stomatal development related genes [58] and in this study, we found that the expression levels of *CqCDK03* and *CqCDK05*, which are homologous to CDK2, also changed significantly under drought stress, it is suggested that these genes may also play a role in quinoa under drought stress by mediating the expression of stomatal development-related genes and affecting stomatal density. The CDKG2 gene confers salt tolerance and promotes flowering in *Arabidopsis thaliana* [28], this gene increased salt tolerance by up-regulating the expression of stress-responsive genes SOS1, SOS2, SOS3, NHX3, RD29B, ABI2, ABI3, MYB15 and P5CS1 [28], and two genes in this study (*CqCDK01* and *CqCDK02*) are homologous to CDKG2, and these two genes also strongly respond to salt stress; It is suggested that these two genes may play a key role in salt stress in a similar way. Zhao’s study showed that [14] CDK2 increased cell division and drought tolerance in *Arabidopsis thaliana* by regulating cycle genes and stomatal development-related genes. CDKF4 in cotton enhances drought and salt tolerance in transgenic plants through reactive oxygen species release and antioxidant levels [48], in this study, we found that the expression levels of two genes belonging to CDKF subfamily (*CqCDK16* and *CqCDK19*) also changed significantly under drought and salt stress. At the same time, we found that the gene pair with the lowest Ka/Ks value (CqCDK07-CqCDK11) showed similar expression pattern under drought and salt stress, and the gene pair with the highest Ka/Ks value (CqCDK02-CqCDK09) showed distinct expression pattern under drought and salt stress, this phenomenon is favorable to support the gene after the replication of functional differences, which is consistent with the study in soybean [68] .On the other hand, transcriptome data suggest that CDK also play important roles in plant responses to other biotic and abiotic stresses. For example, two of the quinoa CDK genes were significantly up-regulated under salt stress, and some of the CqCDK genes were also induced under high temperature and low phosphorus stress. Furthermore, we found that different CqCDK genes differ in response mechanisms to different stresses, implying a diversity of CqCDKs functions in quinoa stress response mechanisms. Our results suggest that most of the CDK genes in quinoa are involved in drought stress and salt stress. These results provide some references for further understanding the function of this family. However, due to the lack of a complete genetic system, quinoa cannot achieve the function of the body gene, so we should urgently improve the quinoa genetic transformation system.

## Conclusion

In summary, we performed a comprehensive and systematic genome-wide analysis of CqCDKs. A total of 22 genes from the quinoa CDK gene family were identified, and bioinformatics analysis and expression profiling of these genes were performed, to determine their potential function in quinoa growth, development and stress response. Expression analysis and functional prediction indicated that CDK gene may play significant and complex roles in regulating plant adaptation to different environmental conditions. It was found that CqCDK genes strongly responded to drought and salt stress. In addition, overexpression of *CqCDK15* gene showed strong resistance to salt stress and drought stress. These results provide important references for further research on the application of CDK gene family in the regulation of plant growth, development and differentiation, and help to elucidate the mechanism of stress resistance in quinoa.

### Electronic supplementary material

Below is the link to the electronic supplementary material.

Supplementary Material 1

Supplementary Material 2

Supplementary Material 3

Supplementary Material 4

Supplementary Material 5

## Data Availability

The reference genome assembly used for data analysis was obtained from National Center for Biotechnology Information (NCBI: GCF_001683475.1). The datasets analysed during this study are included in this published article and its supplementary information files.
