# Supplementary figures and images for: Identification of CDK gene family and functional analysis of CqCDK15 under drought and salt stress in quinoa
Source: BMC Genomics. 2023 Aug 17;24:461. doi: 10.1186/s12864-023-09570-4 (PMC10433607; doi:10.1186/s12864-023-09570-4)

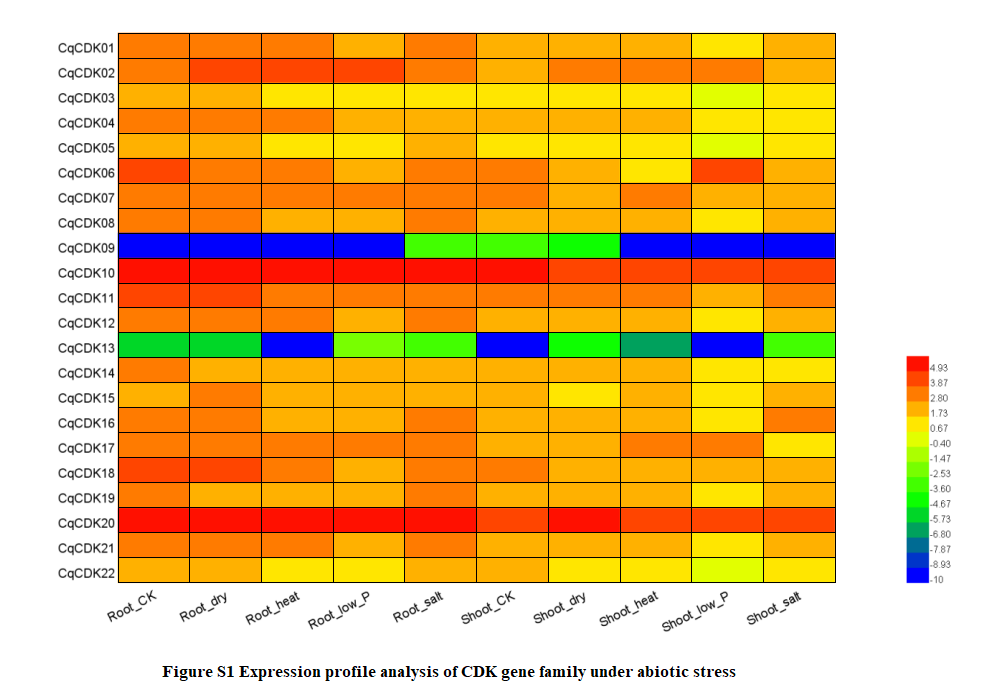

Supplement: Supplementary file 1 — Supplementary Material 1 [file 12864_2023_9570_MOESM1_ESM.png]
