# Supplementary material for: Identification of CDK gene family and functional analysis of CqCDK15 under drought and salt stress in quinoa
Source: BMC Genomics. 2023 Aug 17;24:461. doi: 10.1186/s12864-023-09570-4 (PMC10433607; doi:10.1186/s12864-023-09570-4)
Supplement: Supplementary file 3 — Supplementary Material 3 [file 12864_2023_9570_MOESM3_ESM.docx]

| Gene | Alpha helix | Extended strand | Random coil |
| --- | --- | --- | --- |
| *CqCDK01* | 222（29.40%） | 101（13.38%） | 432（ 57.22%） |
| *CqCDK02* | 129（ 21.83%） | 133（22.50%） | 329（55.67%） |
| *CqCDK03* | 139（ 27.47%） | 84（16.60%） | 283（55.93%） |
| *CqCDK04* | 303（ 37.36%） | 193（23.80%） | 315（38.84%） |
| *CqCDK05* | 129（ 25.49%） | 84（16.60%） | 293（57.91%） |
| *CqCDK06* | 115（37.22%） | 47（15.21%） | 147（47.57%） |
| *CqCDK07* | 80（ 27.78%） | 44（15.28%） | 164（56.94%） |
| *CqCDK08* | 125（27.29%） | 83（18.12%） | 250（54.59%） |
| *CqCDK09* | 68（ 24.03%） | 81（28.62%） | 134（47.35%） |
| *CqCDK10* | 229（36.01%） | 110（17.30%） | 297（46.7%） |
| *CqCDK11* | 87（26.61%） | 53（16.21%） | 187（ 57.19%） |
| *CqCDK12* | 158（33.55%） | 76（16.14%） | 237（50.72%） |
| *CqCDK13* | 96（ 18.90%） | 146（28.74%） | 266（52.36%） |
| *CqCDK14* | 135（ 26.37%） | 77（15.04%） | 300（58.59%） |
| *CqCDK15* | 79（45.66%） | 28（16.18%） | 66（38.15%） |
| *CqCDK16* | 154（32.15%） | 74（15.45%） | 251（52.4%） |
| *CqCDK17* | 154（25.67%） | 132（22.00%） | 314（52.33%） |
| *CqCDK18* | 105（33.98%） | 50（16.18%） | 154（49.84%） |
| *CqCDK19* | 150（ 31.32%） | 70（14.61%） | 259（54.07%） |
| *CqCDK20* | 108（25.78%） | 78（18.62%） | 233（55.61） |
| *CqCDK21* | 237（30.42%） | 100（12.84%） | 442（56.74%） |
| *CqCDK22* | 144（ 34.29%） | 58（13.81%） | 218（51.90%） |

**Table S2 Secondary structure analysis of CDK gene family in Quinoa**
